# Associations between metabolic-inflammatory biomarkers and *Helicobacter pylori* infection: an interpretable machine learning prediction approach

**DOI:** 10.3389/fnut.2025.1674585

**Published:** 2025-11-19

**Authors:** Yue Zhang, Ruifeng Duan, Xin Chen, Lijuan Wei

**Affiliations:** Department of Gastroenterology and Digestive Endoscopy Center, The Second Hospital of Jilin University, Changchun, China

**Keywords:** *Helicobacter pylori*, triglyceride glucose, machine learning, inflammatory index, metabolic

## Abstract

**Background:**

This study investigated the association between metabolic-inflammatory markers and *Helicobacter pylori* (HP) infection using interpretable machine learning models, with a focus on the triglyceride-glucose (TyG) index, TyG/HDL-C ratio, and systemic inflammatory biomarkers.

**Methods:**

Data from 2,924 NHANES participants and 1,021 patients from the Second Hospital of Jilin University were analyzed. Associations between metabolic-inflammatory markers and HP were assessed using multivariable regression. Eleven machine learning models were compared for predictive performance, evaluated by AUC, accuracy, sensitivity, specificity, precision, F1 score, and Kappa statistic. Interpretability was assessed via SHAP values, calibration plots, confusion matrices, and decision curve analysis.

**Results:**

In NHANES, the TyG index was independently associated with HP infection (OR = 1.25, 95% CI 1.06–1.48, *P* = 0.009), and the TyG/HDL-C ratio remained significant after full adjustment (OR = 1.16, 95% CI 1.07–1.25, *P* < 0.001), while SIRI, IBI, and CRP lost significance. In the external Chinese cohort, the TyG association attenuated (*P* = 0.057), but higher TyG/HDL-C quartiles remained significant. Among 11 algorithms, Random Forest (RF) and Gaussian Process (GP) achieved the highest AUCs on the training set (both 0.97) but dropped markedly on the validation set (both 0.75), indicating overfitting. In contrast, XGBoost (XGB) and MLP maintained more consistent AUCs between training (0.77) and validation (0.77), reflecting better generalization. DeLong’s test indicated that both RF and XGB significantly outperformed baseline models (*P* < 0.001), while XGB demonstrated more stable validation performance. Decision curve and SHAP analyses supported the clinical relevance of XGB, highlighting Race and Age as dominant contributors.

**Conclusion:**

The TyG index and TyG/HDL-C ratio were independently associated with HP infection. Among machine learning models, XGBoost demonstrated the most stable and generalizable performance (AUC 0.77 in both training and validation), whereas RF and GP (AUC 0.97 → 0.75) exhibited overfitting. These results suggest that XGB provides a more reliable framework for infection risk prediction, though the cross-sectional design precludes causal inference.

## Background

Insulin resistance (IR), defined as a reduced sensitivity and responsiveness to insulin, is closely associated with cardiovascular diseases through mechanisms involving excessive sympathetic nervous system activation, endothelial dysfunction, and chronic inflammation (1–3). Increasing evidence highlights IR as a central driver of these pathophysiological changes (4). Recent studies have further identified the triglyceride-glucose (TyG) index and its derivatives (e.g., the triglyceride to HDL-C ratio) as reliable surrogate markers for IR (5). Calculated using fasting triglyceride and glucose levels, the TyG index provides a simple and reliable estimate of insulin sensitivity (6).

*Helicobacter pylori* (HP), a Gram-negative bacterium with a global seroprevalence exceeding 50%—particularly prevalent in Asian populations (7) is not only implicated in gastrointestinal diseases (e.g., gastric cancer, chronic gastritis, peptic ulcers), but also in a variety of extra-gastric disorders affecting the nervous and cardiovascular systems (8–10). Growing evidence suggests that HP infection can exacerbate IR by promoting the release of pro-inflammatory cytokines (e.g., IL-6, TNF-α, CRP), activating inflammatory signaling pathways, and inducing systemic chronic inflammatory responses (11–13). Moreover, HP-specific antibodies, such as cytotoxin-associated gene A (CagA), play critical roles in triggering inflammatory cascades and disrupting host metabolic homeostasis (12).

Currently, limited research has investigated the relationship between metabolic-inflammatory markers and HP infection. While elevated TyG levels have been associated with HP seropositivity and increased mortality risk in cross-sectional studies, the role of inflammation as a potential mediator between metabolic dysfunction and HP infection has not yet been explored (14). Moreover, there remains a lack of effective tools for self-assessment of HP infection risk in the general population.

In this study, we hypothesize that metabolic-inflammatory dysregulation may be associated with increased susceptibility to HP infection. To test this hypothesis, we conducted a comprehensive analysis that integrates data from both the NHANES cohort and a Chinese population-based cohort, aiming to: Examine the associations between metabolic-inflammatory indices and HP infection status. Develop a machine learning-based prediction model to facilitate individual-level risk assessment of HP infection. Enable early prevention and intervention strategies through personalized risk profiling. By combining clinical and demographic data with advanced machine learning techniques, this study provides novel insights into the pathophysiological links between metabolic-inflammatory imbalance and HP infection, while offering a practical tool for risk stratification.

## Materials and methods

**FIGURE 1:**
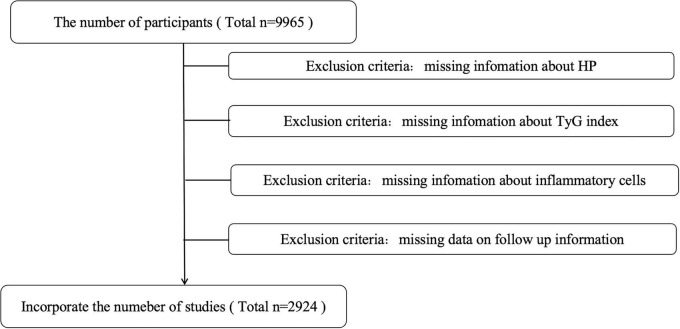
Flowchart of study participants.

This study utilized data from two independent cohorts. The first data Collection and Definitions: This cross-sectional study included 2,924 adult participants from the 1999–2000 cycle of the National Health and Nutrition Examination Survey (NHANES), with data integrated using the unique “SENQ” identifier assigned to each participant. Exclusion criteria are detailed in Figure 1. All analyses accounted for the complex survey design using the survey package in R. Sample weights, primary sampling units (PSUs), and strata were incorporated to ensure national representativeness consistent with U.S. Census Bureau population estimates. The second cohort comprised 1,021 patients from the Second Hospital of Jilin University. Participants were stratified into quartiles based on their TyG index for subgroup analysis. Continuous variables are presented as weighted means (95% confidence intervals) calculated using Taylor series linearization, and categorical variables are reported as weighted proportions. Missing data were addressed through multiple imputation.

The analysis included participants who underwent serological testing for HP and provided fasting blood samples. The NHANES study protocol was approved by the National Center for Health Statistics (NCHS) Research Ethics Review Board, and all participants provided written informed consent.

### Handling of missing and imputed values

Missing data were addressed separately for the two cohorts to reflect differences in sampling design and data completeness:

NHANES cohort: Variables with <10% missingness were imputed using multiple imputation by chained equations (MICE), preserving the complex survey structure. Chinese cohort: Given the smaller sample size and minimal missingness, single imputation was performed using median substitution for continuous variables and mode imputation for categorical variables. Outliers and implausible laboratory values were identified using interquartile range (IQR) inspection and excluded prior to imputation to prevent bias propagation.

This two-tiered strategy ensured data comparability while minimizing distortion of statistical inferences.

### Variables and measurements

Laboratory measurements: Fasting serum glucose and triglycerides were measured at the Mobile Examination Center (MEC). Baseline demographic and clinical characteristics were collected, including age, gender, race, obesity, diabetes, smoking, alcohol consumption, and high cholesterol status. The TyG index was calculated as follows (15):


TyG=ln⁢[Triglycerides⁢(mg/dL)×Glucose⁢(mg/dL)/2]

The triglyceride-to-HDL-C ratio (TG/HDL-C) was computed as:


TG/HDL⁢_⁢C=Triglycerides⁢(mg/dL)/HDL-C⁢(mg/dL)

Inflammatory indices were derived at the MEC using a Beckman Coulter HMX hematology analyzer as follows (16):


SIRI=Monocytes×Neutrophils/Lymphocytes

IBI=CRP×Neutrophils/Lymphocytes

Serum CRP levels were quantified using latex-enhanced nephelometry (BN ProSpec, Siemens Healthcare). In the Chinese dataset, additional markers were included: gallbladder polyps, renal cysts, hepatic cysts, and MASLD.

### Outcome definition

The primary outcome was HP infection, assessed using different diagnostic methods in the two cohorts:

In the NHANES cohort, HP infection status was determined by seropositivity to HP-specific IgG antibodies using standardized laboratory protocols. In the Chinese cohort, HP infection was assessed by the carbon-14 (^1^4C) urea breath test (UBT), which detects active HP colonization by measuring labeled CO2 in the exhaled breath. This test is considered a non-invasive and highly specific clinical gold standard for active infection.

### Statistical analysis

All statistical analyses were conducted in accordance with guidelines provided by the CDC^1^. Given the complex, multistage, stratified probability sampling design of NHANES, analyses incorporated sample weights, clustering, and stratification. Means were used to describe continuous participant characteristics, and proportions were used for categorical variables. Baseline characteristics were described according to TyG quartiles. Homogeneity of variance was tested, followed by Bonferroni *post hoc* comparisons as appropriate.

Weighted binary logistic regression models were employed to assess the associations of TyG, TyG/HDL-C, SIRI, IBI, and CRP indices with HP seropositivity. Regression results were reported as β coefficients and 95% confidence intervals (CI) for both continuous and quartile-based predictor variables. A *P*-value < 0.05 was considered statistically significant.

NHANES cohort: Model 1 was unadjusted. Model 2 was adjusted for age, gender, and race/ethnicity. Model 3 was further adjusted for lifestyle factors (smoking and alcohol use) and cardiometabolic comorbidities obesity, diabetes, HPN, and hypercholesterolemia to evaluate independent associations.

Chinese cohort: Model 1: Unadjusted. Model 2: Adjusted for age and gender. Model 3: Further adjusted for age, gender, and key biochemical/metabolic variables, including: Age, Gender, DM, CHOL, Gallbladder.polyps, Renal.cyst, hepatic.cyst, MASLD.

### Machine learning models and evaluation strategy

All continuous features were standardized to have zero mean and unit variance, while categorical variables were transformed using one-hot encoding. Missing values were initially handled using median imputation, but we acknowledge that this method may introduce bias. To verify robustness, sensitivity analyses using MICE were performed, yielding consistent model rankings. Features with near-zero variance and highly collinear predictors (|r| > 0.9) were excluded prior to modeling. A unified preprocessing pipeline was consistently applied across all models to ensure fair comparability.

To identify the most informative predictors, we employed Recursive Feature Elimination (RFE) with Random Forest (RF) as the base learner. RFE was performed exclusively within the training set to avoid information leakage and was nested inside a 10-fold cross-validation framework, ensuring that feature ranking was recalculated in each resampling fold. Candidate subset sizes of 5, 10, and all available predictors were evaluated. Model performance, measured by the area under the ROC curve (AUC), guided the feature elimination process. Across folds, a stable set of five variables—Race, Age, TyG, IBI, and CRP—consistently emerged as the most discriminative predictors and was retained for subsequent model development.

To optimize predictive performance, we implemented 11 supervised machine learning algorithms, including RF, Extreme Gradient Boosting (XGB), Neural Networks (NN), Multilayer Perceptron (MLP), Logistic Regression (LR), Naïve Bayes (NB), Support Vector Machine (SVM), C5.0, Gradient Boosting Machine (GBM), K-Nearest Neighbors (KNN), and Gaussian Process (GP). These models were chosen for their complementary strengths in handling non-linear relationships, imbalanced data, and high-dimensional feature spaces.

The dataset was divided into 70% training and 30% testing subsets using stratified sampling to preserve outcome distribution. Within the training data, nested cross-validation was employed to tune hyperparameters and evaluate model robustness. Specifically, a 5-fold inner loop combined grid and random search to optimize hyperparameters based on cross-validated AUC, while an outer 10-fold cross-validation assessed model consistency and minimized overfitting.

Finally, pairwise DeLong tests were applied to ROC curves to evaluate statistical differences in model performance, with AUC serving as the primary performance criterion (17, 18).

### Model interpretation and clinical utility

Although RF achieved the highest AUC on the training set, it demonstrated a substantial performance drop on validation, indicating potential overfitting. In contrast, XGB maintained consistent AUC and F1-score across both datasets, supporting its superior generalization and clinical applicability. Therefore, XGB was selected as the final model for interpretation and deployment.

For interpretability, confusion matrices and calibration plots evaluated model reliability, while Decision Curve Analysis (DCA) quantified clinical net benefit against “treat-all” and “treat-none” strategies. SHAP values further elucidated feature contributions, identifying Race and Age as dominant predictors.

## Results

Table 1 presents the baseline characteristics of participants stratified by TyG quartiles (weighted analysis). Table 2 presents the baseline characteristics of the Second Hospital of Jilin University participants stratified by TyG quartile. Several potential confounders associated with HP seropositivity showed significant variation across TyG quartiles, including age, sex, race, diabetes, hypertension, obesity, smoking, and alcohol consumption (*P* < 0.05).

**TABLE 1:** Baseline characteristics according to triglyceride-glucose (TyG) quartiles, weighted.

Variables	Total (*n* = 2,924)	Q1 (*n* = 867)	Q2 (*n* = 723)	Q3 (*n* = 653)	Q4 (*n* = 681)	Statistic	*P*
Age, M (Q1, Q3)	33.00 (17.00, 57.00)	18.00 (15.00, 30.00)	30.00 (17.00, 54.00)	44.00 (23.00, 64.00)	54.00 (36.00, 66.00)	χ^2^ = 651.16#	<0.001
Gender, *n* (%)						χ^2^ = 3.26	0.354
Man	1,495 (51.13)	455 (52.48)	382 (52.84)	324 (49.62)	334 (49.05)	–	–
Woman	1,429 (48.87)	412 (47.52)	341 (47.16)	329 (50.38)	347 (50.95)	–	–
Race, *n* (%)						χ^2^ = 199.71	<0.001
Mexican American	962 (32.90)	255 (29.41)	247 (34.16)	203 (31.09)	257 (37.74)	–	–
Non-Hispanic White	1,083 (37.04)	223 (25.72)	273 (37.76)	290 (44.41)	297 (43.61)	–	–
Non-Hispanic Black	616 (21.07)	309 (35.64)	145 (20.06)	94 (14.40)	68 (9.99)	–	–
Other	263 (8.99)	80 (9.23)	58 (8.02)	66 (10.11)	59 (8.66)	–	–
Marital, *n* (%)						χ^2^ = 263.17	<0.001
No	1,773 (60.64)	695 (80.16)	453 (62.66)	343 (52.53)	282 (41.41)	–	–
Yes	1,151 (39.36)	172 (19.84)	270 (37.34)	310 (47.47)	399 (58.59)	–	–
Education, *n* (%)						χ^2^ = 177.93	<0.001
No	729 (24.93)	101 (11.65)	160 (22.13)	194 (29.71)	274 (40.23)	–	–
Yes	2,195 (75.07)	766 (88.35)	563 (77.87)	459 (70.29)	407 (59.77)	–	–
PIR, *n* (%)						χ^2^ = 9.33	0.025
No	2,304 (78.80)	660 (76.12)	560 (77.46)	530 (81.16)	554 (81.35)	–	–
Yes	620 (21.20)	207 (23.88)	163 (22.54)	123 (18.84)	127 (18.65)	–	–
Drink, *n* (%)						χ^2^ = 92.47	<0.001
No	2,598 (88.85)	833 (96.08)	654 (90.46)	554 (84.84)	557 (81.79)	–	–
Yes	326 (11.15)	34 (3.92)	69 (9.54)	99 (15.16)	124 (18.21)	–	–
Smoke, *n* (%)						χ^2^ = 41.63	<0.001
No	2,524 (86.32)	789 (91.00)	639 (88.38)	548 (83.92)	548 (80.47)	–	–
Yes	400 (13.68)	78 (9.00)	84 (11.62)	105 (16.08)	133 (19.53)	–	–
DM, *n* (%)						χ^2^ = 314.10	<0.001
No	2,731 (93.40)	861 (99.31)	710 (98.20)	623 (95.41)	537 (78.85)	–	–
Yes	193 (6.60)	6 (0.69)	13 (1.80)	30 (4.59)	144 (21.15)	–	–
BP, *n* (%)						χ^2^ = 203.74	<0.001
No	2,333 (79.79)	807 (93.08)	607 (83.96)	462 (70.75)	457 (67.11)	–	–
Yes	591 (20.21)	60 (6.92)	116 (16.04)	191 (29.25)	224 (32.89)	–	–
TC, *n* (%)						χ^2^ = 235.41	<0.001
No	2,556 (87.41)	852 (98.27)	661 (91.42)	543 (83.15)	500 (73.42)	–	–
Yes	368 (12.59)	15 (1.73)	62 (8.58)	110 (16.85)	181 (26.58)	–	–
Obesity, *n* (%)						χ^2^ = 216.85	<0.001
No	2,166 (74.08)	760 (87.66)	580 (80.22)	437 (66.92)	389 (57.12)	–	–
Yes	758 (25.92)	107 (12.34)	143 (19.78)	216 (33.08)	292 (42.88)	–	–
HP, *n* (%)							
No	1,858 (63.54)	624 (71.97)	463 (64.04)	422 (64.62)	349 (51.25)	χ^2^ = 71.44	<0.001
Yes	1,066 (36.46)	243 (28.03)	260 (35.96)	231 (35.38)	332 (48.75)	–	–
CRP, M (Q1, Q3)	0.18 (0.06, 0.43)	0.06 (0.03, 0.20)	0.17 (0.06, 0.40)	0.23 (0.10, 0.55)	0.33 (0.16, 0.65)	χ^2^ = 452.02#	<0.001
TyG/HDL-C, M (Q1, Q3)	2.71 (2.09, 3.53)	2.01 (1.72, 2.40)	2.54 (2.07, 3.06)	3.09 (2.55, 3.66)	4.05 (3.37, 4.96)	χ^2^ = 1410.65#	<0.001
IBI, M (Q1, Q3)	0.35 (0.10, 0.97)	0.12 (0.04, 0.40)	0.31 (0.09, 0.87)	0.47 (0.18, 1.16)	0.66 (0.31, 1.57)	χ^2^ = 441.70#	<0.001
SIRI, M (Q1, Q3)	0.97 (0.67, 1.49)	0.84 (0.55, 1.28)	0.94 (0.67, 1.50)	1.08 (0.71, 1.57)	1.11 (0.82, 1.63)	χ^2^ = 106.04#	<0.001

#Kruskal-waills test. χ^2^, Chi-square test; M, median; Q1, 1st quartile; Q3, 3rd quartile.

**TABLE 2:** Baseline characteristics according to triglyceride-glucose (TyG) quartiles.

Variables	Total (*n* = 1,021)	1 (*n* = 255)	2 (*n* = 163)	3 (*n* = 347)	4 (*n* = 256)	Statistic	*P*
Age, M (Q1, Q3)	42.00 (34.00, 52.00)	36.00 (30.00, 46.00)	42.00 (35.00, 52.00)	43.00 (34.00, 53.00)	45.00 (37.00, 54.00)	χ^2^ = 55.35#	<0.001
Gender, *n* (%)						χ^2^ = 64.43	<0.001
Man	574 (56.22)	101 (39.61)	83 (50.92)	200 (57.64)	190 (74.22)	–	–
Woman	447 (43.78)	154 (60.39)	80 (49.08)	147 (42.36)	66 (25.78)	–	–
DM, *n* (%)						χ^2^ = 93.32	<0.001
No	977 (95.69)	255 (100.00)	163 (100.00)	341 (98.27)	218 (85.16)	–	–
Yes	44 (4.31)	0 (0.00)	0 (0.00)	6 (1.73)	38 (14.84)	–	–
CHOL, *n* (%)						χ^2^ = 28.42	<0.001
No	909 (89.03)	233 (91.37)	149 (91.41)	322 (92.80)	205 (80.08)	–	–
Yes	112 (10.97)	22 (8.63)	14 (8.59)	25 (7.20)	51 (19.92)	–	–
Gallbladder polyps, *n* (%)						χ^2^ = 21.69	<0.001
No	751 (73.56)	169 (66.27)	136 (83.44)	243 (70.03)	203 (79.30)	–	–
Yes	270 (26.44)	86 (33.73)	27 (16.56)	104 (29.97)	53 (20.70)	–	–
Gallstone, *n* (%)						χ^2^ = 3.86	0.276
No	936 (91.67)	234 (91.76)	154 (94.48)	311 (89.63)	237 (92.58)	–	–
Yes	85 (8.33)	21 (8.24)	9 (5.52)	36 (10.37)	19 (7.42)	–	–
Surgery, *n* (%)						-	0.055
No	1,005 (98.43)	255 (100.00)	160 (98.16)	339 (97.69)	251 (98.05)	–	–
Yes	16 (1.57)	0 (0.00)	3 (1.84)	8 (2.31)	5 (1.95)	–	–
Kidney stone, *n* (%)						χ^2^ = 4.46	0.215
No	982 (96.18)	248 (97.25)	160 (98.16)	329 (94.81)	245 (95.70)	–	–
Yes	39 (3.82)	7 (2.75)	3 (1.84)	18 (5.19)	11 (4.30)	–	–
Renal cyst, *n* (%)						χ^2^ = 17.33	<0.001
No	939 (91.97)	246 (96.47)	154 (94.48)	304 (87.61)	235 (91.80)	–	–
Yes	82 (8.03)	9 (3.53)	9 (5.52)	43 (12.39)	21 (8.20)	–	–
Hepatic cyst, *n* (%)						χ^2^ = 23.54	<0.001
No	896 (87.76)	232 (90.98)	143 (87.73)	282 (81.27)	239 (93.36)	–	–
Yes	125 (12.24)	23 (9.02)	20 (12.27)	65 (18.73)	17 (6.64)	–	–
MASLD, *n* (%)						χ^2^ = 109.77	<0.001
No	437 (42.80)	149 (58.43)	80 (49.08)	168 (48.41)	40 (15.62)	–	–
Yes	584 (57.20)	106 (41.57)	83 (50.92)	179 (51.59)	216 (84.38)	–	–
HP, *n* (%)						χ^2^ = 57.06	<0.001
No	427 (41.82)	148 (58.04)	79 (48.47)	132 (38.04)	68 (26.56)	–	–
Yes	594 (58.18)	107 (41.96)	84 (51.53)	215 (61.96)	188 (73.44)	–	–
SIRI quantile, M (Q1, Q3)	3.00 (2.00, 4.00)	2.00 (1.00, 3.00)	3.00 (1.00, 4.00)	2.00 (2.00, 3.00)	3.00 (2.00, 4.00)	χ^2^ = 18.02#	<0.001
TyG/HDL-C quantile, M (Q1, Q3)	3.00 (2.00, 4.00)	1.00 (1.00, 2.00)	2.00 (1.00, 3.00)	3.00 (3.00, 3.00)	4.00 (3.00, 4.00)	χ^2^ = 591.58#	<0.001

#Kruskal-waills test. χ^2^, Chi-square test; -, Fisher exact; M, median, Q1, 1st quartile, Q3, 3rd quartile.

### Association between metabolic-inflammatory markers and HP IgG seropositivity

Table 3 presents the results of the multivariable regression analysis examining the association between metabolic-inflammatory markers and *H. pylori* (HP) seropositivity (IgG antibodies). In the unadjusted model (Model 1), the TyG index demonstrated a significant positive association with HP infection (OR = 1.66, 95% CI: 1.48–1.86; *P* < 0.001). This association remained significant but was slightly attenuated in Model 2, which was adjusted for age, gender, smoking, alcohol consumption, and race (OR = 1.17, 95% CI: 1.01–1.36; *P* = 0.039). The association persisted in Model 3, which included additional adjustments for high cholesterol, obesity, diabetes, and hypertension, though the effect size was further reduced (OR = 1.25, 95% CI: 1.06–1.48; *P* = 0.009).

**TABLE 3:** Multivariable logistic regression analysis for *Helicobacter pylori* (HP) infection risk (β, 95% CI) National Health and Nutrition Examination Survey (NHANES).

Variables	Model 1		Model 2		Model 3	
	OR (95% CI)	*P*	OR (95% CI)	*P*	OR (95% CI)	*P*
TyG (continuous)	1.66 (1.48∼1.86)	<0.001	1.17 (1.01∼1.36)	0.039	1.25 (1.06∼1.48)	0.009
TyG (quartile)						
Q1	1.00 (reference)	–	1.00 (reference)	–	1.00 (reference)	–
Q2	1.44 (1.17∼1.78)	<0.001	1.16 (0.91∼1.47)	0.237	1.17 (0.92∼1.49)	0.203
Q3	1.41 (1.13∼1.75)	0.002	0.92 (0.71∼1.20)	0.554	0.97 (0.74∼1.27)	0.820
Q4	2.44 (1.98∼3.02)	<0.001	1.36 (1.04∼1.78)	0.026	1.46 (1.09∼1.96)	0.010
TyG/HDL-C (continuous)	1.24 (1.17∼1.32)	<0.001	1.12 (1.04∼1.21)	0.002	1.16 (1.07∼1.25)	<0.001
TyG/HDL-C (quartile)						
Q1	1.00 (reference)	–	1.00 (reference)	–	1.00 (reference)	–
Q2	1.17 (0.94∼1.44)	0.156	1.08 (0.85∼1.36)	0.543	1.09 (0.86∼1.39)	0.465
Q3	1.47 (1.18∼1.82)	<0.001	1.27 (1.00∼1.63)	0.051	1.34 (1.05∼1.72)	0.020
Q4	1.94 (1.56∼2.40)	<0.001	1.32 (1.02∼1.70)	0.035	1.42 (1.08∼1.85)	0.011
SIRI (continuous)	0.91 (0.84∼0.99)	0.046	0.90 (0.81∼1.00)	0.052	0.90 (0.82∼1.00)	0.057
SIRI (quartile)						
Q1	1.00 (reference)	–	1.00 (reference)	–	1.00 (reference)	–
Q2	1.00 (0.81∼1.23)	0.972	0.89 (0.70∼1.13)	0.325	0.89 (0.70∼1.13)	0.342
Q3	0.98 (0.79∼1.20)	0.817	0.83 (0.65∼1.06)	0.138	0.84 (0.66∼1.07)	0.163
Q4	0.90 (0.73∼1.11)	0.329	0.85 (0.66∼1.08)	0.185	0.85 (0.67∼1.09)	0.210
IBI (continuous)	1.01 (0.98∼1.04)	0.481	0.97 (0.94∼1.00)	0.055	0.97 (0.94∼1.00)	0.073
IBI (quartile)						
Q1	1.00 (reference)	–	1.00 (reference)	–	1.00 (reference)	–
Q2	1.42 (1.14∼1.76)	0.002	1.05 (0.82∼1.35)	0.678	1.07 (0.83∼1.38)	0.592
Q3	1.70 (1.38∼2.11)	<0.001	0.97 (0.75∼1.25)	0.800	0.99 (0.76∼1.29)	0.956
Q4	1.71 (1.39∼2.10)	<0.001	0.86 (0.66∼1.12)	0.263	0.89 (0.68∼1.17)	0.404
CRP (continuous)	1.20 (1.08∼1.34)	<0.001	0.97 (0.86∼1.09)	0.576	0.98 (0.87∼1.10)	0.720
CRP (quartile)						
Q1	1.00 (reference)	–	1.00 (reference)	–	1.00 (reference)	–
Q2	1.22 (0.98∼1.52)	0.081	0.79 (0.62∼1.02)	0.075	0.80 (0.62∼1.03)	0.089
Q3	1.71 (1.38∼2.13)	<0.001	0.99 (0.76∼1.28)	0.936	1.01 (0.78∼1.32)	0.917
Q4	1.78 (1.45∼2.19)	<0.001	0.79 (0.61∼1.02)	0.069	0.81 (0.61∼1.07)	0.133

OR, odds ratio; CI, confidence interval. Model 1: Crude. Model 2: Adjust: Age, Gender, Drink, Smoke, Race. Model 3: Adjust: Age, Gender, Drink, Smoke, Race, High cholesterol, Obesity, Diabetes, HTN.

The TyG/HDL-C ratio, treated as a continuous variable, consistently showed a robust positive association with HP seropositivity across all models (Model 3: OR = 1.16, 95% CI: 1.07–1.25; *P* < 0.001). When the TyG/HDL-C ratio was categorized into quartiles, the highest quartile (Q4) demonstrated a strong positive association in the crude model (OR = 1.94, *P* < 0.001), but the association weakened in the adjusted models (Model 2: OR = 1.32, *P* = 0.035; Model 3: OR = 1.42, *P* = 0.011). No significant associations were observed for Q2 in any of the models.

In contrast, SIRI showed a significant positive association with HP infection in the crude model (OR = 0.91, *P* = 0.046), but the association was no longer significant in the adjusted models or when categorized into quartiles. IBI and CRP exhibited similar patterns, with significant associations in the crude model but a lack of significance after adjustment and in quartile-based analysis.

Table 4 presents the associations between HP infection and metabolic-inflammatory markers in patients from the Second Hospital of Jilin University. In the crude model (Model 1), the TyG index (continuous) showed a significant positive association with HP infection (OR = 2.35, 95% CI: 1.85–2.98; *P* < 0.001). The association remained significant but was attenuated in the adjusted models. In Model 2, adjusted for age and gender, the odds ratio decreased (OR = 1.90, 95% CI: 1.48–2.42; *P* < 0.001), and in Model 3, further adjusted for diabetes, cholesterol levels, gallbladder polyps, renal cysts, hepatic cysts, and MASLD, the association was no longer significant (OR = 1.32, 95% CI: 0.99–1.75; *P* = 0.057).

**TABLE 4:** Multivariable logistic regression analysis for *Helicobacter pylori* (HP) infection risk (β, 95% CI) (China).

Variables	Model 1		Model 2		Model 3	
	OR (95% CI)	*P*	OR (95% CI)	*P*	OR (95% CI)	*P*
TyG (continuous)	2.35 (1.85∼2.98)	<0.001	1.90 (1.48∼2.42)	<0.001	1.32 (0.99∼1.75)	0.057
TyG (quartile)						
Q1	1.00 (reference)	–	1.00 (reference)	–	1.00 (reference)	–
Q2	1.47 (0.99∼2.18)	0.056	1.25 (0.83∼1.88)	0.278	1.17 (0.74∼1.86)	0.491
Q3	2.25 (1.62∼3.13)	<0.001	1.81 (1.29∼2.56)	<0.001	1.72 (1.17∼2.53)	0.005
Q4	3.82 (2.63∼5.55)	<0.001	2.71 (1.83∼4.01)	<0.001	1.53 (0.97∼2.40)	0.066
TyG/HDL-C (continuous)	1.61 (1.39∼1.86)	<0.001	1.42 (1.23∼1.65)	<0.001	1.15 (0.98∼1.35)	0.098
TyG/HDL-C (quartile)						
Q1	1.00 (reference)	–	1.00 (reference)	–	1.00 (reference)	–
Q2	1.99 (1.36∼2.92)	<0.001	1.69 (1.14∼2.50)	0.009	1.43 (0.92∼2.21)	0.108
Q3	3.21 (2.28∼4.53)	<0.001	2.44 (1.71∼3.49)	<0.001	2.25 (1.51∼3.37)	<0.001
Q4	3.86 (2.67∼5.58)	<0.001	2.86 (1.94∼4.22)	<0.001	1.78 (1.15∼2.74)	0.010
SIRI (continuous)	1.45 (1.03∼2.03)	0.031	1.33 (0.96∼1.84)	0.087	1.18 (0.86∼1.63)	0.305
SIRI (quartile)						
Q1	1.00 (reference)	–	1.00 (reference)	–	1.00 (reference)	–
Q2	1.86 (1.31∼2.65)	<0.001	1.64 (1.14∼2.37)	0.008	2.14 (1.40∼3.25)	<0.001
Q3	1.65 (1.16∼2.34)	0.005	1.48 (1.03∼2.13)	0.034	1.24 (0.83∼1.87)	0.294
Q4	1.92 (1.35∼2.74)	<0.001	1.64 (1.14∼2.37)	0.008	1.47 (0.98∼2.23)	0.065

OR, odds ratio; CI, confidence interval. Model 1: Crude. Model 2: Adjust: Age, Gender. Model 3: Adjust: Age, Gender, DM, CHOL, Gallbladder.polyps, Renal.cyst, hepatic.cyst, MASLD.

For TyG quartiles, the highest quartile (Q4) exhibited a strong positive association in Model 1 (OR = 3.82, *P* < 0.001), but this association weakened in Model 2 (OR = 2.71, *P* < 0.001) and became non-significant in Model 3 (OR = 1.53, *P* = 0.066). The Q3 quartile showed a significant positive association in all models (Model 1: OR = 2.25, *P* < 0.001; Model 2: OR = 1.81, *P* < 0.001; Model 3: OR = 1.72, *P* = 0.005), while Q2 did not show a significant association in any of the models (*P*-values ranging from 0.056 to 0.491).

The TyG/HDL-C ratio (continuous) also demonstrated a positive association with HP infection, with a strong effect in the crude model (OR = 1.61, 95% CI: 1.39–1.86; *P* < 0.001) and Model 2 (OR = 1.42, 95% CI: 1.23–1.65; *P* < 0.001). However, this association was not significant in Model 3 (OR = 1.15, 95% CI: 0.98–1.35; *P* = 0.098). For TyG/HDL-C quartiles, the highest quartile (Q4) showed a strong positive association in the crude model (OR = 3.86, *P* < 0.001), which decreased but remained significant in Model 2 (OR = 2.86, *P* < 0.001), and was still significant in Model 3 (OR = 1.78, *P* = 0.010). The second (Q2) and third (Q3) quartiles showed varying results, with Q3 showing a significant association in Model 2 (OR = 2.44, *P* < 0.001) and Model 3 (OR = 2.25, *P* < 0.001), while Q2 had a weaker or non-significant association across models.

### Machine learning

For SIRI, the continuous variable showed a significant positive association with HP infection in the crude model (OR = 1.45, 95% CI: 1.03–2.03; *P* = 0.031), but this association became weaker and non-significant in the adjusted models (Model 2: OR = 1.33, *P* = 0.087; Model 3: OR = 1.18, *P* = 0.305). In SIRI quartiles, the second quartile (Q2) showed a significant positive association in all models (Model 1: OR = 1.86, *P* < 0.001; Model 2: OR = 1.64, *P* = 0.008; Model 3: OR = 2.14, *P* < 0.001). The third quartile (Q3) was significant in Model 1 (OR = 1.65, *P* = 0.005) and Model 2 (OR = 1.48, *P* = 0.034), but not in Model 3 (OR = 1.24, *P* = 0.294). The fourth quartile (Q4) also showed a significant association in the crude and adjusted models (Model 1: OR = 1.92, *P* < 0.001; Model 2: OR = 1.64, *P* = 0.008), but the association became borderline significant in Model 3 (OR = 1.47, *P* = 0.065).

**FIGURE 2:**
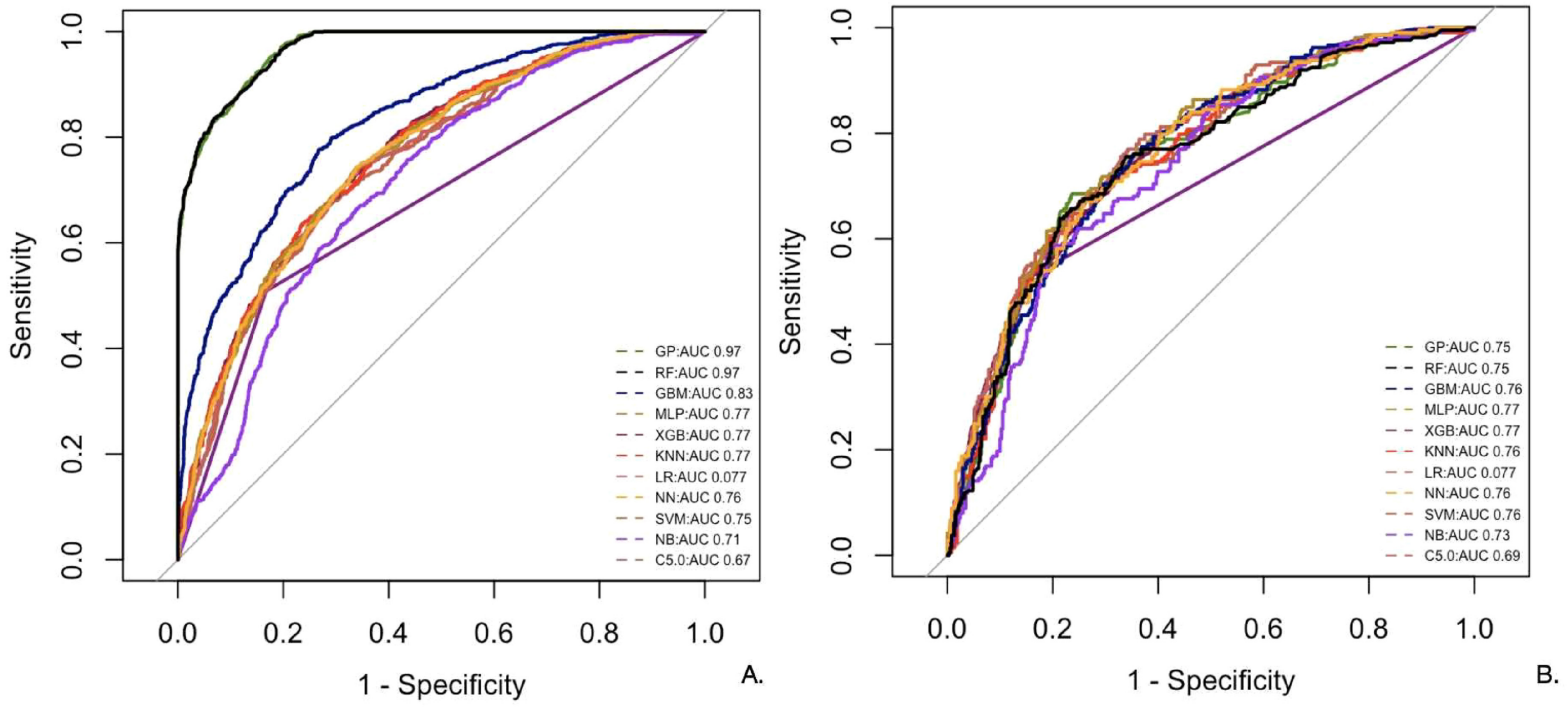
Receiver operating characteristic curves for 11 models. **(A,B)** (training and testing) Display the receiver operating characteristic (ROC) curves of *Performance of 11 machine-learning models* on the training and test datasets: ROC curves. Models include NN, SVM, MLP, GBM, LR, NB, XGB, C5.0, GP, KNN, and RF. The horizontal axis is 1-Specificity, and the vertical axis is Sensitivity. The closer the curve is to the upper left corner, the better the model’s classification performance.

RF and GP performed the best on the training dataset, with RF achieving an AUC of 0.97, Recall of 0.71, and Precision of 0.95, while GP showed similar performance (Table 5 and Figure 2). However, both models exhibited significant drops in performance on the test dataset (AUC: 0.75), indicating a potential overfitting risk due to capturing noise in the training data (Supplementary Table 1). In contrast, XGB demonstrated more consistent performance across both datasets, with strong Precision (0.66) and F1 (0.56), making it a more reliable model for generalization. To assess statistical significance between model performances, pairwise DeLong tests were conducted on ROC curves. These comparisons (Supplementary Table 2) showed that both RF and XGB significantly outperformed baseline models (*P* < 0.001). Notably, RF and XGB differed significantly from each other (*P* < 0.001), while differences between other top models (e.g., RF vs. GP, XGB vs. MLP) were not statistically significant.

**TABLE 5:** Model performance table for 11 models.

Model	AUC	Recall	Precision	Accuracy	Sensitivity	Specificity	F1	Kappa
NN	0.77	0.51	0.65	0.72	0.51	0.84	0.57	0.37
SVM	0.75	0.51	0.64	0.72	0.51	0.84	0.56	0.36
MLP	0.77	0.55	0.64	0.72	0.55	0.82	0.59	0.38
GBM	0.83	0.55	0.72	0.76	0.55	0.88	0.62	0.45
LR	0.77	0.44	0.69	0.72	0.44	0.88	0.53	0.35
NB	0.71	0.44	0.58	0.70	0.44	0.82	0.50	0.27
XGB	0.77	0.48	0.67	0.72	0.48	0.86	0.56	0.37
C5.0	0.67	0.51	0.64	0.71	0.51	0.83	0.57	0.36
GP	0.97	0.70	0.96	0.88	0.70	0.98	0.80	0.72
KNN	0.77	0.45	0.68	0.72	0.45	0.88	0.54	0.35
RF	0.97	0.71	0.95	0.85	0.71	0.98	0.82	0.73

AUC, area under the curve; F1, harmonized average of precision and recall rates; Kappa, the agreement between the predictions of the classification model and the randomized prediction results.

**FIGURE 3:**
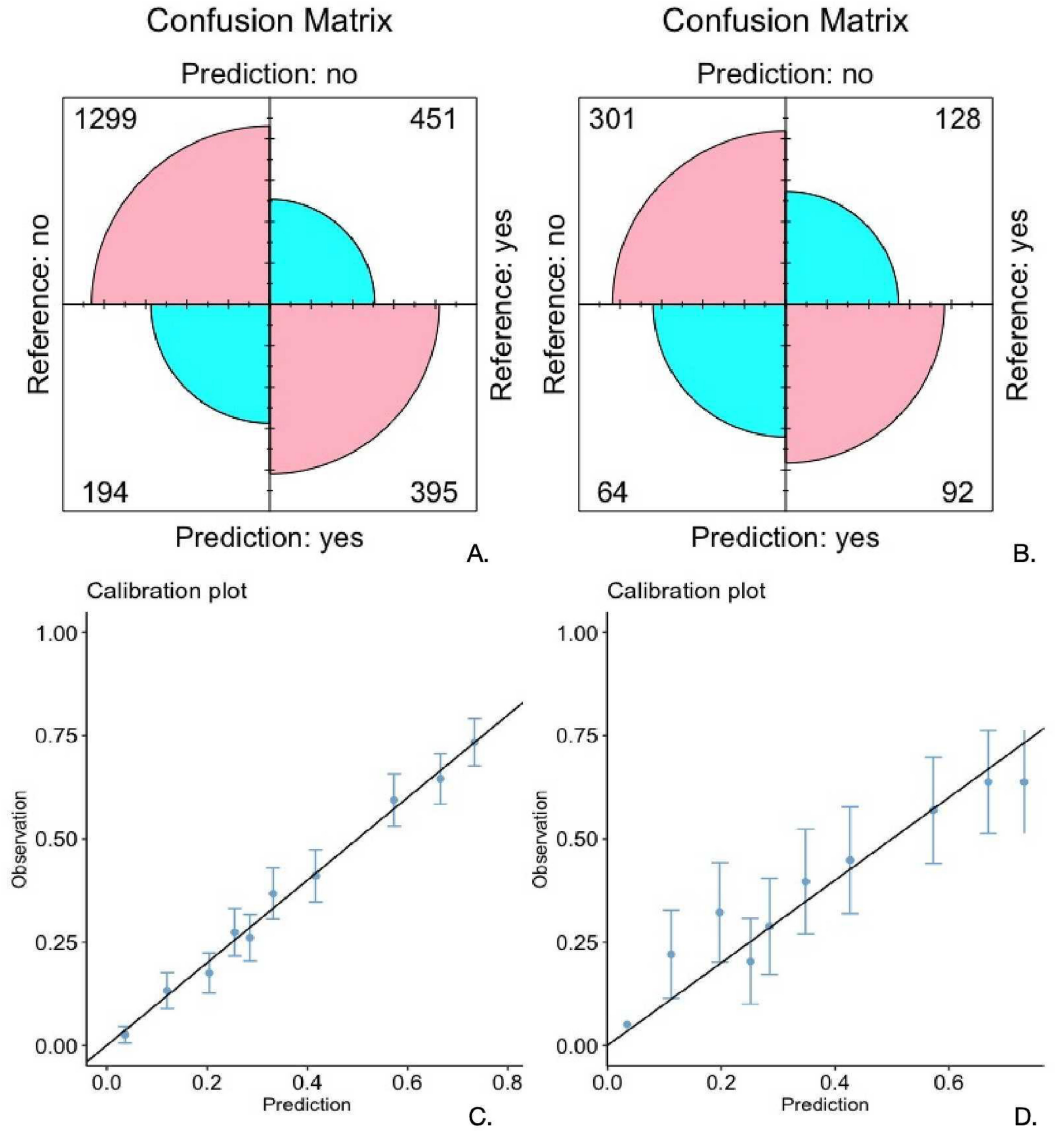
**(A,B)** (training and testing) Display the confusion matrices of the extreme gradient boosting (XGB) model. **(C,D)** (training and testing) Show the calibration plots of the XGB model. The horizontal axis (X-axis) represents the probability of illness predicted by the model, and the vertical axis (Y-axis) represents the actual observed proportion of illness.

Figure 3 illustrates the performance of the XGB model through confusion matrices (Figures 3A, B) and calibration plots (Figures 3C, D) for the training and validation datasets, respectively.

Figures 3A, B present the confusion matrices for the training and validation datasets, respectively. In the training set (Figure 3A), the model correctly identified 1,299 true negatives and 395 true positives, with 194 false positives and 451 false negatives. In the validation set (Figure 3B), the model correctly classified 301 true negatives and 92 true positives, while misclassifying 64 false positives and 128 false negatives. Compared to the training set, the performance in the validation set demonstrates a notable decrease in sensitivity and overall classification accuracy, indicating potential overfitting of the model to the training data (Figure 3).

Figures 3C, D the calibration plots for the training and validation sets, respectively. The X-axis represents the predicted probability of disease, and the Y-axis reflects the observed event frequency. In the training set (Figure 3C), the calibration curve closely aligns with the ideal diagonal line, indicating good agreement between predicted probabilities and actual outcomes. In contrast, the calibration plot for the validation set (Figure 3D) shows increased deviation from the diagonal line and broader confidence intervals, suggesting reduced calibration performance and increased predictive uncertainty when applied to external data (Figure 3).

We used DCA to assess clinical utility of the XGB model. In DCA, the threshold probability (p_*t*_) is the minimum predicted risk at which a clinician would act; it encodes the trade-off between false negatives and false positives [relative harm ≈ p_*t*_/(1−p_*t*_)]. Thus, the range 0.10–0.50 corresponds to clinically plausible scenarios where the harm of an unnecessary intervention is between 1:9 and 1:1 compared with missing a true case—i.e., typical “moderate-risk” decisions such as initiating preventive therapy or ordering confirmatory testing.

**FIGURE 4:**
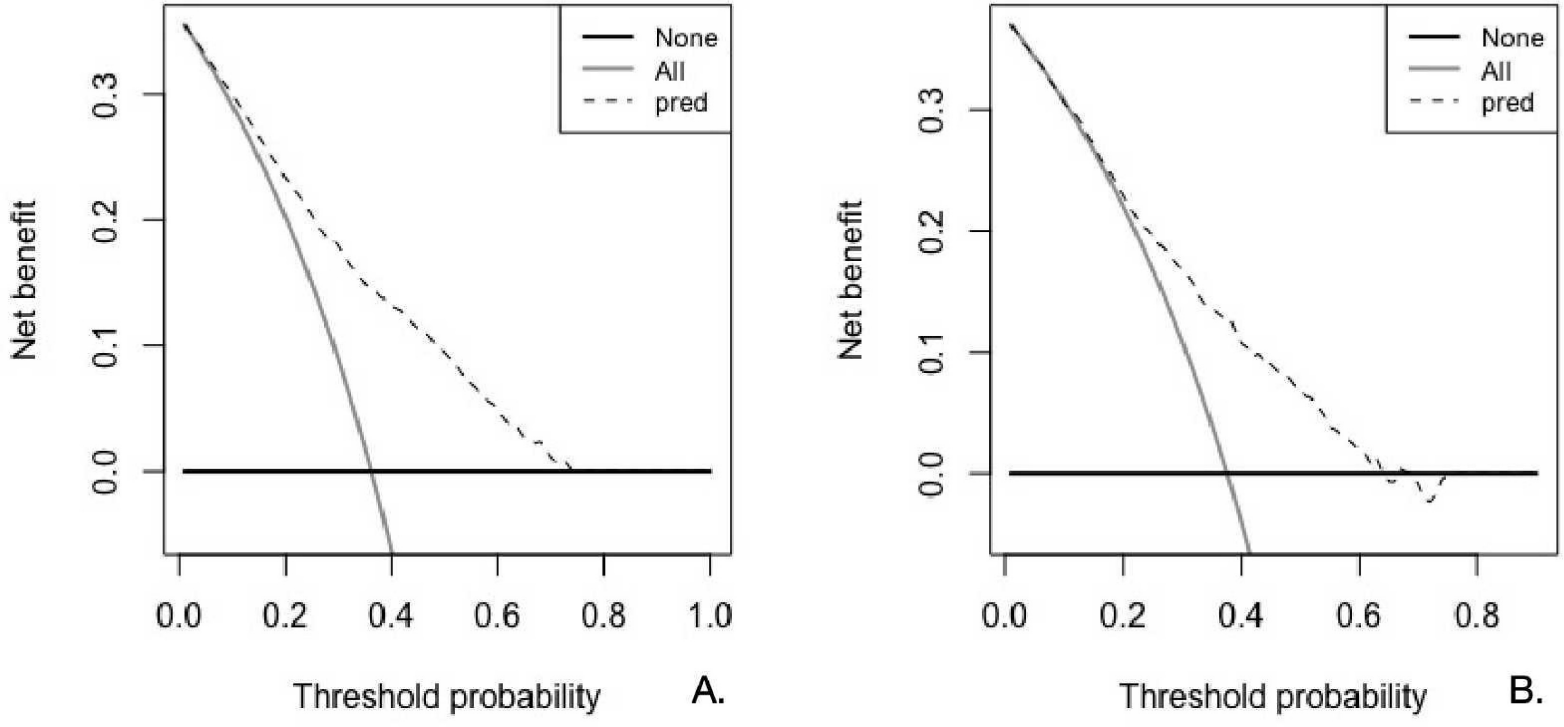
Decision curve analysis (DCA) of the extreme gradient (XGB) model. **(A)** Displays the decision curve for the training set, while **(B)** shows the decision curve for the testing set. The horizontal axis (X-axis) represents the threshold probability—i.e., the predicted probability above which a subject is classified as positive and considered for intervention. The vertical axis (Y-axis) denotes the net benefit, which accounts for the trade-off between true positives and false positives at each threshold. The “All” line represents a strategy in which all patients are assumed to receive treatment, regardless of risk, while the “None” line reflects a strategy where no patients receive treatment. A model is considered clinically useful if it yields a higher net benefit than both the “All” and “None” strategies across a reasonable range of threshold probabilities.

As shown in Figure 4, the XGB curve lies above both reference strategies—Treat-None and Treat-All—through 0.10–0.50 on the training set (Figure 4A), and remains superior through approximately 0.10–0.60 on the validation set (Figure 4B), though with a smaller margin. Practically, if a clinician’s action threshold falls in these ranges, using XGB would yield greater net benefit than existing default strategies, implying fewer unnecessary interventions for a similar or higher number of detected true cases.

**FIGURE 5:**
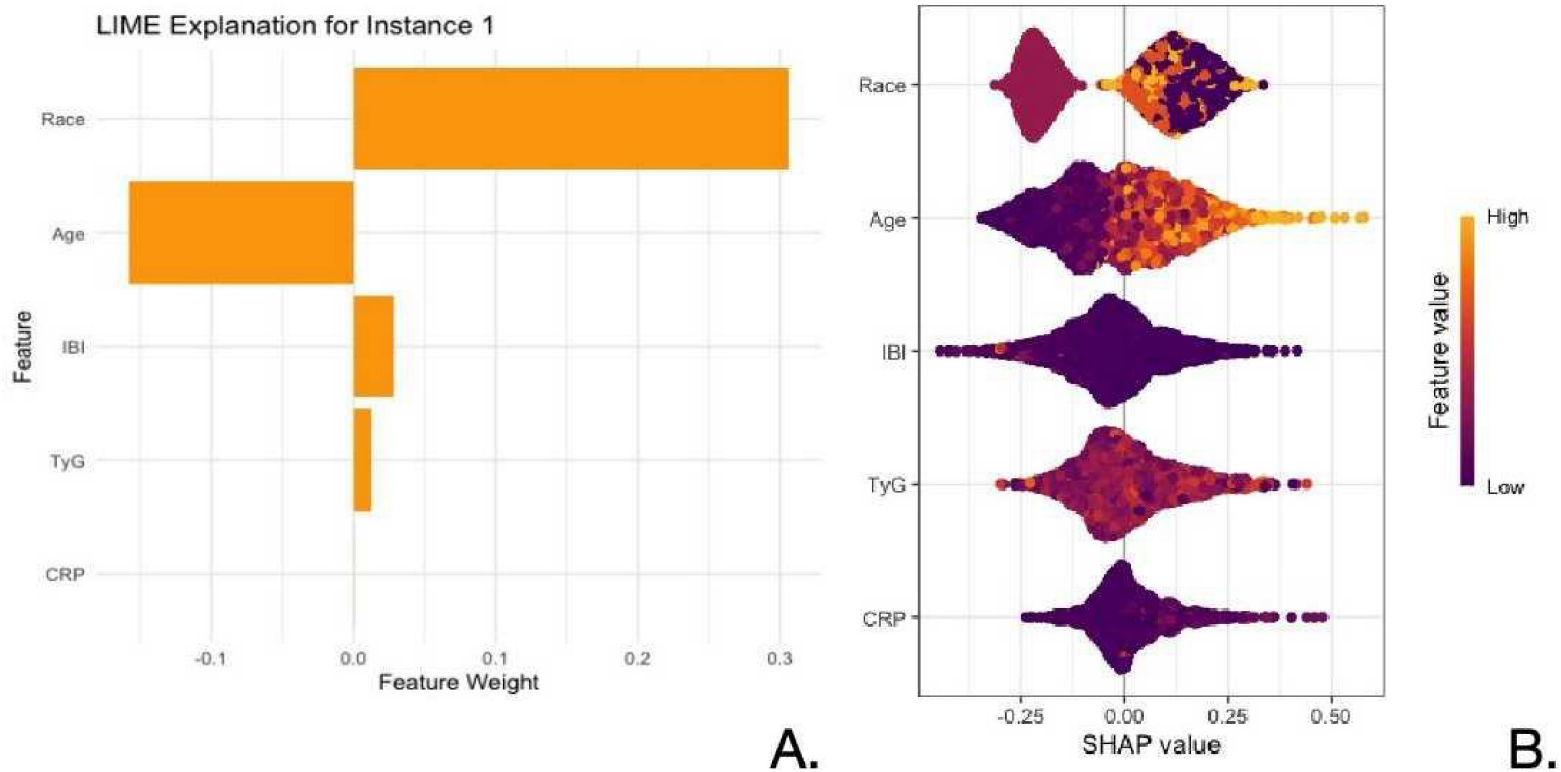
Feature importance and SHapley additive exPlanations (SHAP) values. **(A)** LIME explanation showing the feature weights for each attribute that contributed to the model’s prediction. **(B)** SHAP summary plot, displaying the distribution of SHAP values for each feature across all instances. Color bar: represents the feature values from low (purple) to high (yellow) for each feature.

In this study, we used two interpretability methods, LIME and SHAP, to analyze the feature importance and their impact on the model’s prediction for a given instance (Figure 5).

Figure 5A revealed that, for the most influential features were Race and Age. Race had a substantial positive impact on the prediction with a feature weight of approximately +0.3, indicating that this feature significantly contributed to the positive prediction. Age also played an important role, contributing positively but with a smaller weight compared to Race. Other features such as IBI, TyG, and CRP showed minimal contributions, with TyG and CRP having near-zero or slightly negative weights. These results suggest that for this specific instance, Race and Age were the primary driving factors influencing the model’s prediction.

Figure 5B provided a broader view of the feature importance across all instances. Race emerged as the most influential feature, with a wide SHAP value distribution, indicating its significant and varying impact on the model’s predictions across different samples. Age was also a critical feature, with higher ages (indicated by yellow color) pushing the prediction toward positive outcomes. IBI and TyG had moderate effects, while CRP showed the least impact. The SHAP values demonstrated that Race and Age are the key features in driving the model’s output, with Race being the dominant factor.

## Discussion

This study examined the associations between metabolic–inflammatory markers and HP infection using data from two distinct populations: the NHANES cohort and a Chinese hospital-based sample. Our findings provide new insights into the metabolic–inflammatory dysregulation underlying HP infection and illustrate how interpretable machine learning can help uncover potential biological and contextual patterns related to infection risk.

Insulin resistance is increasingly recognized as a metabolic component linking chronic inflammation and infection. The TyG index, a validated surrogate for IR, has been associated with several chronic diseases, including non-alcoholic fatty liver disease, diabetes, and kidney dysfunction (19–22). Prior studies have also reported positive correlations between HP antibody levels and the TyG index (23). In our analysis, the TyG/HDL-C ratio consistently showed a positive association with HP infection in both cohorts, even after adjusting for demographic and metabolic confounders, supporting its role as a robust indicator of metabolic stress and inflammation.

HP infection is known to trigger chronic gastritis and systemic inflammation through the release of proinflammatory cytokines such as IL-1, IL-6, IL-8, and TNF-α (24–26). These inflammatory cascades disrupt metabolic homeostasis, contributing to insulin resistance, lipid abnormalities, and cardiovascular comorbidities. Elevated TyG index values—reflecting higher triglyceride-glucose burden—often coincide with increased inflammatory activity and greater cardiometabolic risk (16). In contrast, SIRI, IBI, and CRP demonstrated weaker and inconsistent associations, suggesting that traditional inflammatory markers may be more influenced by demographic or environmental factors than by direct metabolic pathways related to HP infection.

Our study aligns with these recommendations by combining interpretable machine learning with validated biochemical indices, promoting both predictive insight and reproducible inference in metabolic–infectious disease research. Such integrative frameworks could clarify how nutrient patterns and metabolic stress jointly shape systemic inflammation and infection susceptibility, advancing both precision nutrition and infection prevention strategies.

Differences between the NHANES and Chinese cohorts underscore the importance of contextual factors. The attenuation of associations in the Chinese cohort may reflect variations in diet, HP strain distribution, healthcare access, or socioeconomic determinants. Interpretability analyses (SHAP and LIME) identified race and age among the most influential predictors of HP infection. However, the role of race in this context should not be interpreted as a biological determinant. Instead, it likely captures social, dietary, and environmental disparities—including differences in sanitation, nutrition, healthcare access, and socioeconomic status—that co-vary with self-reported racial categories. This perspective aligns with recent findings that HP seroprevalence varies among U.S. racial and ethnic groups largely due to environmental and socioeconomic factors (27). This framing aligns with growing consensus in epidemiologic research that race serves as a contextual proxy for social and environmental exposures, rather than reflecting innate biological susceptibility. Recognizing this distinction is critical for preventing misinterpretation of statistical associations and for guiding public-health interventions that address structural and lifestyle factors influencing infection risk (28, 29).

Feature selection via Recursive Feature Elimination (RFE) identified Age, Race, TyG, IBI, and CRP as key predictors (Supplementary Table 3). Among 11 algorithms, Random Forest (RF) and Gaussian Process (GP) achieved high training performance (AUC = 0.97) but showed substantial overfitting in validation (AUC = 0.75). XGBoost (XGB), by contrast, maintained consistent AUCs (0.77 in both sets), demonstrating better generalization and potential clinical applicability. DeLong tests confirmed that both RF and XGB significantly outperformed baseline models (*P* < 0.001), while RF and XGB differed significantly (*P* < 0.001), indicating that XGB provided superior discriminative stability.

These findings support prior work [e.g., Wang et al. (29)] suggesting that integrating interpretable ML approaches with clinical biomarkers can bridge mechanistic understanding and predictive modeling, helping to generate hypotheses about how metabolic–inflammatory pathways and social context interact to influence infection risk.

Given the global burden of HP infection, our findings highlight the translational potential of using TyG-based indices—derived from routine biochemical tests—as cost-effective markers for risk stratification. The XGB model provided meaningful clinical benefit across realistic decision thresholds (0.1–0.5), supporting its utility for targeted screening in primary care or community settings. When embedded in electronic health systems, such models could enable personalized infection risk estimation and guide resource allocation in high-prevalence or low-resource regions.

Notably, most conventional AI and machine learning models for HP diagnosis have relied on endoscopic or histopathological image analysis to identify mucosal lesions and infection patterns. In contrast, our approach uses basic demographic information and metabolic–inflammatory indices to predict infection risk, enabling a non-invasive, low-cost, and generalizable diagnostic alternative suitable for population-level screening (30, 31). Such models may complement imaging-based AI systems by providing biochemical and physiological insights into host–pathogen interactions. Furthermore, chronic HP infection and its eradication therapy can markedly influence nutritional and metabolic status by altering the gastrointestinal microenvironment. Multiple trials have examined how HP eradication affects body weight, BMI, blood pressure, nutritional markers, and lipid profiles (including total cholesterol, LDL-C, HDL-C, and triglycerides), as well as serological nutrition biomarkers such as apolipoproteins (ApoC-II, ApoC-III) (32). These findings remain inconsistent but collectively suggest that HP infection may impact nutrient absorption and systemic metabolism, thereby influencing long-term health outcomes and even life expectancy.

Our study aligns with current recommendations advocating for rigorous, interpretable AI models combined with validated biochemical indices to enhance reproducibility in metabolic–infectious disease research. Such integrative frameworks could clarify how diet-related metabolic stress and inflammation jointly shape infection susceptibility, advancing precision prevention and individualized metabolic assessment strategies.

## Limitations and future directions

This study has several limitations. The cross-sectional design precludes causal inference regarding the directionality between metabolic alterations and HP infection. Despite multivariable adjustment, residual confounding from unmeasured variables (e.g., diet, HP strain, genetics) may persist. Moreover, the external validation was performed in a single-center cohort with limited sample size, which may restrict generalizability. Future longitudinal, multi-ethnic studies enriched with environmental and behavioral data are needed to test the temporal and mechanistic hypotheses proposed here. Additionally, improving model calibration across populations will enhance predictive reliability and clinical applicability.

## Conclusion

In summary, our findings suggest that metabolic–inflammatory dysregulation, reflected by the TyG index and TyG/HDL-C ratio, is significantly associated with HP infection across populations. These results advance the hypothesis that metabolic abnormalities and systemic inflammation jointly contribute to infection susceptibility. By integrating interpretable machine learning with epidemiologic analyses, this study offers a data-driven framework for hypothesis generation regarding metabolic–immune interactions in chronic infection. While causality cannot be inferred, the consistency of associations across cohorts supports the potential utility of metabolic–inflammatory markers for personalized risk assessment. Future longitudinal and mechanistic studies are warranted to validate these hypotheses and explore the bidirectional relationship between metabolic dysfunction and HP infection.

## Data Availability

The raw data supporting the conclusions of this article will be made available by the authors, without undue reservation.

## Footnotes

1
https://wwwn.cdc.gov/nchs/nhanes/tutorials/default.aspx
