# Supplementary material for: Associations between metabolic-inflammatory biomarkers and Helicobacter pylori infection: an interpretable machine learning prediction approach
Source: Front Nutr. 2025 Nov 19;12:1674585. doi: 10.3389/fnut.2025.1674585 (PMC12673668; doi:10.3389/fnut.2025.1674585)
Supplement: Supplementary file 1 [file Table_1.docx]

Supplementary File Table 1:Model performance table for 11 models .

| Model | AUC | Recall | Precision | Accuracy | Sensitivity | Specificity | F1 | Kappa |
| --- | --- | --- | --- | --- | --- | --- | --- | --- |
| NN | 0.76 | 0.50 | 0.65 | 0.72 | 0.50 | 0.84 | 0.56 | 0.36 |
| SVM | 0.76 | 0.54 | 0.68 | 0.74 | 0.54 | 0.85 | 0.60 | 0.41 |
| MLP | 0.77 | 0.55 | 0.64 | 0.72 | 0.55 | 0.82 | 0.59 | 0.38 |
| GBM | 0.76 | 0.48 | 0.63 | 0.71 | 0.48 | 0.84 | 0.55 | 0.34 |
| LR | 0.77 | 0.44 | 0.69 | 0.72 | 0.44 | 0.89 | 0.54 | 0.35 |
| NB | 0.73 | 0.50 | 0.63 | 0.71 | 0.50 | 0.83 | 0.55 | 0.34 |
| XGB | 0.77 | 0.48 | 0.66 | 0.72 | 0.48 | 0.86 | 0.56 | 0.36 |
| C5.0 | 0.69 | 0.53 | 0.66 | 0.73 | 0.53 | 0.85 | 0.59 | 0.53 |
| GP | 0.75 | 0.50 | 0.65 | 0.72 | 0.50 | 0.85 | 0.57 | 0.36 |
| KNN | 0.76 | 0.43 | 0.68 | 0.72 | 0.43 | 0.88 | 0.53 | 0.34 |
| RF | 0.75 | 0.50 | 0.66 | 0.72 | 0.50 | 0.85 | 0.57 | 0.37 |
| AUC, area under the curve; F1, harmonized average of precision and recall rates; Kappa, the agreement between the predictions of the classification model and the randomized prediction results; | | | | | | | | |

Supplementary File Table 2: The result of the Delong test

|  | P value |
| --- | --- |
| RF vs SVM | <0.001 |
| RF vs GBM | <0.001 |
| RF vs C5.0 | <0.001 |
| RF vs GP | 0.697 |
| RF vs KNN | <0.001 |
| RF vs LR | <0.001 |
| RF vs MLP | <0.001 |
| RF vs NB | <0.001 |
| RF vs NN | <0.001 |
| RF vs XGB | <0.001 |
| SVM vs GBM | <0.001 |
| SVM vs C5.0 | <0.001 |
| SVM vs GP | <0.001 |
| SVM vs KNN | <0.001 |
| SVM vs LR | 0.13 |
| SVM vs MLP | <0.001 |
| SVM vs NB | <0.001 |
| SVM vs NN | <0.001 |
| SVM vs XGB | <0.001 |
| GBM vs C5.0 | <0.001 |
| GBM vs GP | <0.001 |
| GBM vs KNN | <0.001 |
| GBM vs LR | <0.001 |
| GBM vs MLP | <0.001 |
| GBM vs NB | <0.001 |
| GBM vs NN | <0.001 |
| GBM vs XGB | <0.001 |
| C5.0 vs GP | <0.001 |
| C5.0 vs KNN | <0.001 |
| C5.0 vs LR | <0.001 |
| C5.0 vs MLP | <0.001 |
| C5.0 vs NB | <0.001 |
| C5.0 vs NN | <0.001 |
| C5.0 vs XGB | <0.001 |
| GP vs KNN | <0.001 |
| GP vs LR | <0.001 |
| GP vs MLP | <0.001 |
| GP vs NB | <0.001 |
| GP vs NN | <0.001 |
| GP vs XGB | <0.001 |
| KNN vs LR | <0.001 |
| KNN vs MLP | 0.281 |
| KNN vs NB | <0.001 |
| KNN vs NN | 0.183 |
| KNN vs XGB | 0.627 |
| LR vs MLP | 0.022 |
| LR vs NB | <0.001 |
| LR vs NN | 0.003 |
| LR vs XGB | 0.002 |
| MLP vs NB | <0.001 |
| MLP vs NN | 0900 |
| MLP vs XGB | 0.499 |
| NB vs NN | <0.001 |
| NB vs XGB | <0.001 |
| NN vs XGB | 0.377 |

Supplementary File Table 3 Variables Tested in Association and Prediction Analyses

| Category | Variable | Description/Role in Analysis | Association Outcome |
| --- | --- | --- | --- |
| Demographic Factors | Race | Self-reported race/ethnicity; contextual proxy for socio-environmental factors | Significant predictor; high SHAP importance |
|  | Age | Continuous variable; biological and exposure-related risk | Significant predictor across models |
| Metabolic Markers | TyG | ln\[fasting triglycerides (mg/dL) × fasting glucose (mg/dL)/2]; surrogate for insulin resistance | Significant association in NHANES; attenuated after adjustment in Chinese cohort |
|  | TyG/HDL-C | TyG index divided by HDL cholesterol; reflects metabolic–inflammatory burden | Robust, consistent positive association across cohorts |
| Inflammatory Markers | SIRI | (Neutrophils × Monocytes) / Lymphocytes | Inconsistent; significance lost after adjustment |
|  | IBI | (Neutrophils × Monocytes) / (Lymphocytes + Hemoglobin) | Retained as top predictive feature despite limited direct association |
| Socioeconomic Factors | Education, Income, BMI, Smoking, Alcohol use | Covariates in adjusted models | Controlled for confounding |
| Machine Learning Predictors (Top 5 via RFE) | Race, Age, TyG, IBI, CRP | Most discriminative subset used for model development | Used in RF, XGB, and GP models |
